# A 10-year follow-up of infliximab monotherapy for refractory uveitis in Behçet’s syndrome

**DOI:** 10.1038/s41598-020-78718-z

**Published:** 2020-12-17

**Authors:** Noe Horiguchi, Koju Kamoi, Shintaro Horie, Yuko Iwasaki, Hisako Kurozumi-Karube, Hiroshi Takase, Kyoko Ohno-Matsui

**Affiliations:** 1 Department of Ophthalmology & Visual Science, Graduate School of Medical and Dental Sciences, Tokyo Medical and Dental University (TMDU), 1-5-45 Yushima, Bunkyo-ku, Tokyo, 113-8519 Japan; 2 Department of Ophthalmology, Nagano Prefectural Federation of Agricultural Cooperatives for Health and Welfare, Saku General Hospital, Nagano, Japan

**Keywords:** Eye diseases, Uveal diseases

## Abstract

Infliximab (IFX) was the first biologic introduced for refractory uveitis treatment in Behçet’s syndrome (BS). However, there have been few reports on the safety and efficacy of IFX monotherapy over follow-up periods of more than 10 years. This retrospective study evaluated the 10-year safety and efficacy of IFX monotherapy compared to IFX combination therapies with colchicine or corticosteroid for refractory uveitis in BS patients. Monotherapy was performed in 30 eyes of 16 patients while combination therapies were performed in 20 eyes of 11 patients. Continuation of IFX occurred in 70.3% of enrolled patients for 10 years without any significant difference noted in the retention rate between the monotherapy and combination therapies (p = 0.86). Reduction of ocular inflammatory attacks and improvement of best corrected visual acuity occurred in the monotherapy group after 10 years, which was equivalent to that for the combination therapies. Although adverse events (AEs) or therapy discontinuation occurred during the initial 5 years in both therapies, no AEs were observed for either therapy after 6 years. Our results suggested that IFX monotherapy proved to be effective and not inferior to combination therapies over a 10-year follow-up. Although loss of response and AEs may be noticed during the initial 5-year period, a safe and effective continuation can be expected thereafter.

## Introduction

Behçet’s syndrome (BS) is a multisystemic inflammatory disease characterized by recurrent oral aphthous ulcers, genital ulcers, skin lesions, and uveitis. In addition to these main features, BS may affect multiple organs including cutaneous, articular, neurological, intestinal, and pulmonary systems ^1^. Recurrent severe uveitis leads to irreversible severe vision loss ^2,3^. According to the medical treatment recipient certificates issued in 2014, there were 20,035 people who suffered from BS in Japan ^4^.

A survey undertaken in the early 2000s in Japan to examine BS patients found that there was poor visual acuity even though these patients had been treated with colchicine (COL), corticosteroid (CS), and immunosuppressant agents such as cyclosporin A (CsA) ^5^. These results suggested that conventional anti-inflammatory drugs might not be all that effective in suppressing uveitis in BS patients.

Infliximab (IFX), which is an anti-TNF-α antibody, was first approved for use worldwide and administered for the treatment of severe uveitis in BS patients in Japan in 2007 ^6^. To date, a high effectiveness of IFX for BS treatment has been reported in both Japan and in other countries ^6–12^. However, there have been few reports on the safety and efficacy of IFX over follow-up periods of more than 10 years ^13^. Furthermore, as the long-term efficacy and safety of IFX monotherapy has yet to be definitively clarified, it remains unknown as to whether concomitant drugs should be administered during IFX treatments.

In conjunction with the drug restrictions and the guidelines for BS treatment in Japan^14^, COL, CS, and CsA are permitted and recommended as anti-inflammatory drugs for use in BS patients for the purpose of seizure suppression. Therefore, IFX is generally given to BS patients after failed treatments with these other drugs. Furthermore, due to the huge financial burden for BS patients who need to regularly use IFX^15–17^, it has been estimated that worldwide, IFX is administered after encountering the ineffectiveness of these other conventional anti-inflammatory agents.

Taken together with the current issues mentioned above, information on the long-term follow-up after IFX administration would be valuable for ophthalmologists with regard to real-world clinical practices for the treatment of BS. Therefore, the present study reports on our experience regarding the long-term efficacy and safety of IFX in the treatment of BS-related uveitis, with a particular focus on the potential differences between monotherapy and combination therapy.

## Results

A total of 50 eyes of 27 consecutive patients met the inclusion criteria, while 4 eyes with permanent blindness before IFX treatment were excluded. All patients showed bilateral uveitis. Table 1 lists the clinical features, including ophthalmologic characteristics/extraocular manifestations, and criteria fulfillment of the Behçet’s Disease Research Committee of Japan (BRCJ), International Study Group (ISG), and International Criteria for Behçet’s Disease (ICBD).

**Table 1:** Clinical features and criteria fulfillment.

**Ocular characteristics**			
Anterior uveitis	0	/50	0%
Intermediate uveitis	0	/50	0%
Posterior uveitis	3	/50	6.0%
Panuveitis	47	/50	94.0%
Retinal vasculitis	48	/50	96.0%
**Extraocular characteristics**			
Mucosal involvement	27	/27	100%
Skin involvement	24	/27	88.9%
Articular involvement	4	/27	14.8%
CNS involvement	2	/27	7.4%
GI involvement	5	/27	18.5%
**Criteria fulfillment**			
BRCJ	27	/27	100%
ISG	24	/27	88.9%
ICBD	27	/27	100%

CNS: central nervous system, GI: gastrointestinal, BRCJ: Behçet’s Disease Research Committee of Japan, ISG: International Study Group, ICBD: International Criteria for Behçet’s Disease.

Male-to-female ratio was 23:4, with a mean age at the start of IFX of 37.3 ± 12.1 (22–76) years old. Follow-up term was 10.1 ± 1.0 years for the overall enrolled patients, with a mean duration of IFX administration of 7.6 ± 4.1 years. Table 2 presents the details of the previous conventional anti-inflammatory treatments administered prior to the initiation of IFX.

**Table 2:** Details of anti-inflammatory drugs before IFX therapies.

**Previous treatment before IFX monotherapy**			
CS	1	/16	6.3%
CsA	7	/16	43.8%
COL	5	/16	31.3%
Other	0	/16	0.0%
**Previous treatment before IFX co-administered with CS**			
CS	8	/8	100.0%
CsA	4	/8	50.0%
COL	2	/8	25.0%
Other	0	/8	0.0%
**Previous treatment before IFX co-administered with COL**			
CS	0	/3	0.0%
CsA	0	/3	0.0%
COL	2	/3	66.7%
Other	0	/3	0.0%

IFX: infliximab, CS: corticosteroid, COL: colchicine, CsA: cyclosporin A.

IFX treatment details included initiation of IFX monotherapy in 30 eyes of 16 patients and initiation as a combination therapy in 20 eyes of 11 patients. Details of the combination therapies included administration of IFX with CS (11.1 ± 2.6 mg/day) in 8 patients and administration of IFX with COL (0.83 ± 0.29 mg/day) in 3 patients. None of the patients were administered IFX with CsA (Table 3).

**Table 3:** Details of IFX monotherapy and combination therapies.

Therapy	Cases	Dose	
**Monotherapy**			
IFX monotherapy	16 cases		
**Combination therapy**			
IFX + CS	8 cases		
mg/day (range)		11.1 ± 2.6	(7.5–1.5)
IFX + COL	3 cases		
mg/day (range)		0.83 ± 0.29	(0.5–1.0)
IFX + CsA	0 cases		

IFX: infliximab, CS: corticosteroid, COL: colchicine, CsA: cyclosporin A.

### Retention rate of IFX therapies

**Figure 1:**
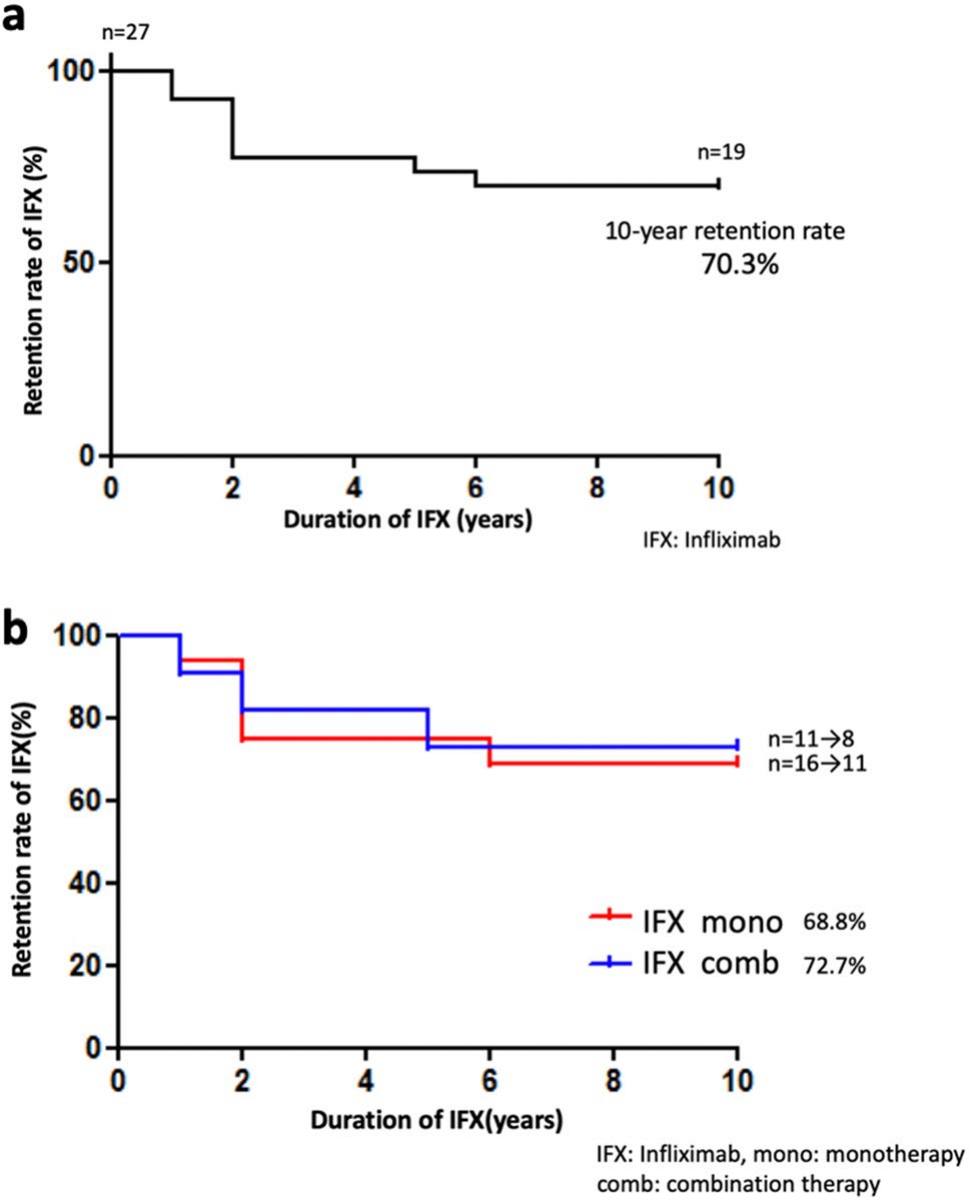
Retention rate of IFX therapies. (**a**) The overall 10-year retention rate of IFX therapy in patients was 70.3%. (**b**) The 10-year retention rate of IFX monotherapy was 68.8%, whereas that for the combination therapy was 72.7%. There was no significant difference between the monotherapy and combination therapy (p = 0.86).

For the IFX retention rate, IFX was discontinued in 8 out of 27 enrolled patients during the 10-year treatment period. The timing and the number of discontinued patients were as follows: 2 patients dropped out within 1 year, 4 patients within 2 years, 1 patient within 4 years, and 1 patient within 5 years. Overall analysis showed there were no patients who discontinued IFX after 6 continuous years, with 70.3% of these patients able to continue IFX treatments for 10 years (Fig. 1a). As seen in Fig. 1b, there was no significant difference for the retention rate at the 10-year point between the IFX monotherapy (68.4%) and combination therapies (72.7%) (p = 0.86).

### Frequency of ocular inflammatory attacks

**Figure 2:**
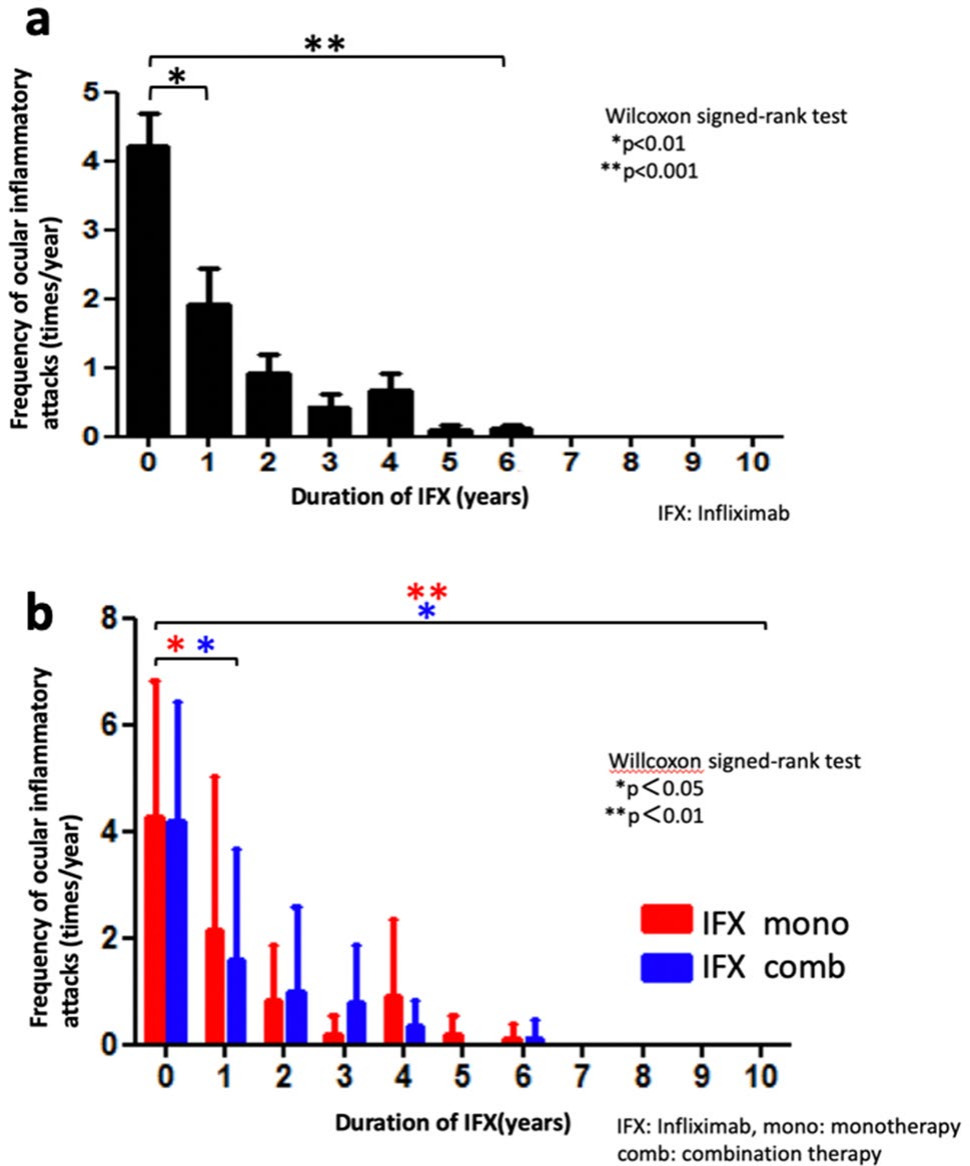
Frequency of inflammatory attacks. (**a**) The overall mean frequency of inflammatory attacks was 4.2 times/year before IFX therapy. Inflammatory attacks significantly decreased to 1.9 times/year at 1 year (p = 0.0030). A slight increase in the attacks (0.7 times/year) was seen at 4 years. Inflammatory attacks were not seen after 7 years of IFX treatment. (**b**) A comparison of the frequency of inflammatory attacks for the IFX monotherapy and combination therapies showed that the inflammatory attacks were significantly decreased by each of the therapies during the 10-year follow-up. In monotherapy, an increase of inflammatory attacks (0.9 times/year) was seen at 4 years. A slight increase in the inflammatory attacks was seen at 6 years in the combination therapy.

Ocular inflammatory attack was defined as any type of acute episodes of intraocular inflammation including with iritis, hypopyon, chorioretinitis, retinal vasculitis, retinal vein occlusion, optic neuritis, retinal neovascularization and vitreous hemorrhage, which was in line with that previously reported ^18,19^. Overall, the mean frequency of inflammatory attacks was 4.2 times/year before IFX therapy. Inflammatory attacks significantly decreased to 1.9 times/year at 1 year (p = 0.0030), 0.9 times/year at 2 years, and 0.4 times/year at 3 years. However, there was a slight increase of attacks (0.7 times/year) seen at 4 years. Subsequently, attacks decreased to 0.1 times/year at 5 years and 0.1 times/year at 6 years (p = 0.0001). Inflammatory attacks were not observed after 7 years of IFX treatment (Fig. 2a).

Comparison of the frequency of inflammatory attacks between the IFX monotherapy and combination therapy, inflammatory attacks before the IFX administration of IFX monotherapy and combination therapy were 4.3 and 4.2 times/year, respectively. At 1 year, there was a decrease of attacks seen for the IFX monotherapy (2.1 times/year p = 0.047), and for the combination therapy (1.6 times/year p = 0.028). At 4 years in the monotherapy group, there was an increase in the inflammatory attacks (0.9 times/year) as compared to the previous year (0.2 times/year). In combination therapy, there was a slight increase in the inflammatory attacks seen at 6 years (0.1 times/year) as compared to the previous 5 years (0 times/year). These inflammatory attacks were not observed after 7 years of continuous treatment, with this suppression status maintained thereafter. A comparison between before and the point after 10 years of continuous treatment demonstrated there was a suppression of inflammatory attacks during this time period (monotherapy: p = 0.0037, combination therapy: p = 0.014) (Fig. 2b).

### Best corrected visual acuity (BCVA)

**Figure 3:**
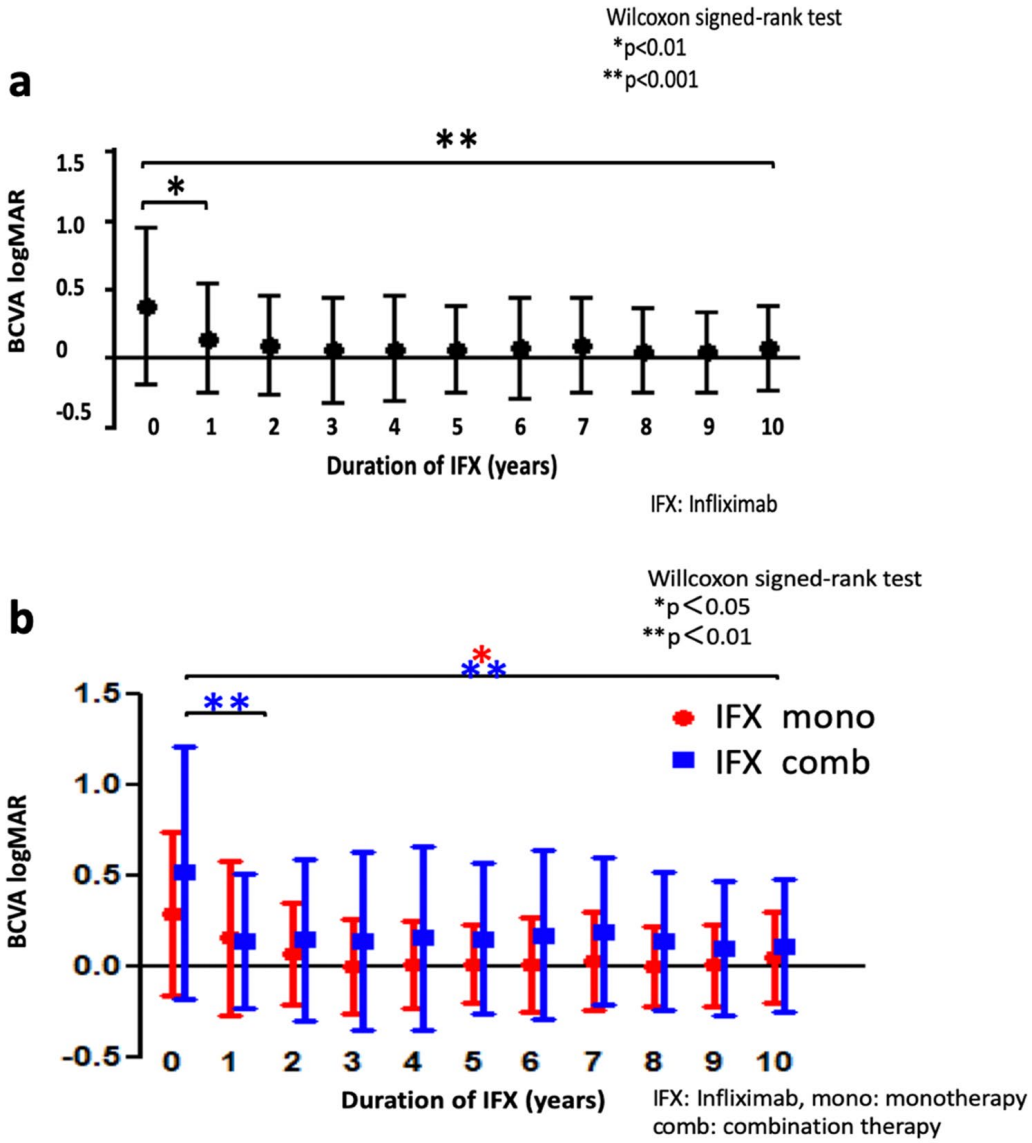
Best corrected visual acuity (BCVA). (**a**) The overall mean BCVA for the patients was 0.38 before IFX administration, with a significant improvement to 0.15 seen at 1 year (p = 0.0016), which was well maintained thereafter. (**b**) A comparison of the BCVA between the monotherapy and combination therapies found there was improvement in both groups after 1 year of IFX treatment (monotherapy: p = 0.058, combination therapy: p = 0.004), with the efficacy subsequently prolonged after that.

Overall, the mean BCVA for the patients was 0.38 before IFX administration, with a significant improvement to 0.15 at 1 year (p = 0.0016) with the BCVA maintained thereafter. Mean BCVA was 0.07 at 10 years (p = 0.0003) (Fig. 3a). A comparison of the monotherapy and combination therapy before and after the administration of IFX showed that the BCVA improved in both groups after 1 year of IFX treatment (monotherapy: p = 0.058, combination therapy: p = 0.004) with the efficacy shown to be continued to be maintained after the initial treatment period. BCVA improved at 10 years as compared to the values found prior to the IFX administration (mono: p = 0.017, combination therapy: p = 0.0097) (Fig. 3b).

### CS-sparing effect

**Figure 4:**
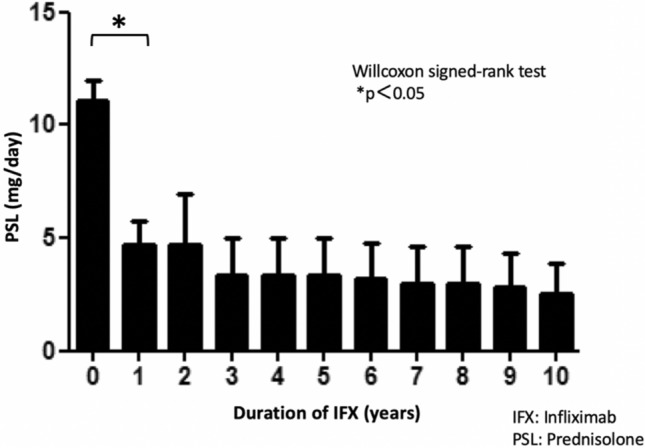
Corticosteroid (CS)-sparing effect. The analysis of the 10-year CS-sparing effect demonstrated that the steroid-sparing effect reached -8.6 mg/day (11.1 mg/day to 2.5 mg/day: 77.5%). There were 2 patients that were able to withdraw from CS during this period.

The CS used in this study was prednisolone, with the mean value of the daily dose of prednisolone shown in Fig. 4. The CS-sparing effect analysis examined 8 patients who were co-administered CS at the time of IFX administration, with a mean CS dose of 11.1 mg/day. Mean CS dose significantly decreased to 4.7 mg/day in 1 year (p = 0.02). Mean CS doses at 3, 6, and 9 years were 3.3, 3.2, and 2.8 mg/day respectively. After the 10-year IFX treatment, the mean CS dose in 6 patients who could continue the IFX treatment (2 patients discontinued IFX) was 2.5 mg/day. This demonstrates that the steroid-sparing effect reached -8.6 mg/day (77.5%). Complete tapering off of CS was achieved in 3 patients during the 10-year follow-up. An increase in CS during follow-up was seen in 2 patients. In conjunction with dropping out of the IFX treatment, there were 2 patients who were withdrawn from the CS (Fig. 4).

### Occurrence and timing of adverse events (AEs)

There were 11 AEs seen in 10 patients during the 10-year follow-up. An infusion reaction was the most frequent AE (8 cases), while 2 out of the 11 AEs were classified as serious events, such as military tuberculosis and bacterial pneumonia (Table 4). The overall rate of severe AEs in the monotherapy group was 6.3% (1 of 16 cases) with an infusion reaction observed in 31.25% (5 of 16 cases). The overall rate of severe AEs in the combination therapy was 9.1% (1 of 11 cases), with an infusion reaction in 27.3% (3 of 11 cases).

**Table 4:** Details of AEs.

AEs	Number	Therapy
**Severe**		
Military tuberculosis	1 case	Mono
Bacterial pneumonia	1 case	Comb (CS)
**Other**		
Infusion reaction	8 cases	
	5 cases	Mono
	2 cases	Comb (CS)
	1 case	Comb (COL)
Psoriasis	1 case	Mono

Mono: monotherapy, Comb: combination therapy, AEs: adverse events, IFX: infliximab, CS: corticosteroid, COL: colchicine.

Table 5 shows the data for the discontinued patients. Reasons for discontinuation were as follows: infection (2 patients: bacterial pneumonia and military tuberculosis), infusion reaction (2 patients), lack of efficacy (1 patient), loss of efficacy (1 patient), and paradoxical reaction (1 patients: psoriasis). In 1 patient, IFX was discontinued and the patient was switched to adalimumab (ADA) due to an outbreak of intestinal Behçet’s syndrome. The treatment types that resulted in discontinuation were monotherapy in 37.5% (6 of 16 patients) and combination therapy (CS) in 25% (2 of 8 patients). In the cases in which IFX continuation was achieved, the patients suffered AEs of a slight skin reaction at the infusion site. Resolution of the slight skin reaction was achieved via the co-administration of d-chlorpheniramine maleate (2 cases), epinastine hydrochloride (3 cases), and the co-administration of both agents (1 case).

**Table 5:** Details of IFX discontinuation.

Age range	Sex	Treatment	Reason for discontinuation	Duration (months)	Treatment after discontinuation of IFX
60 s	M	Comb (CS)	Bacterial pneumonia	19	CS
40 s	M	Mono	Military tuberculosis	1	COL
30 s	F	Mono	Infusion reaction	16	CsA
30 s	M	Mono	Infusion reaction	55	CsA
30 s	M	Comb (CS)	Lack of efficacy	0.3	CS,COL
20 s	M	Mono	Loss of efficacy	16	CsA
30 s	M	Mono	Psoriasis	23	COL
70 s	M	Mono	Intestinal BS	61	Adalimumab

BD: Behçet’s syndrome, Mono: monotherapy, Comb: combination therapy, IFX: infliximab, CS: corticosteroid, COL: colchicine, CsA: cyclosporin A.

**Figure 5:**
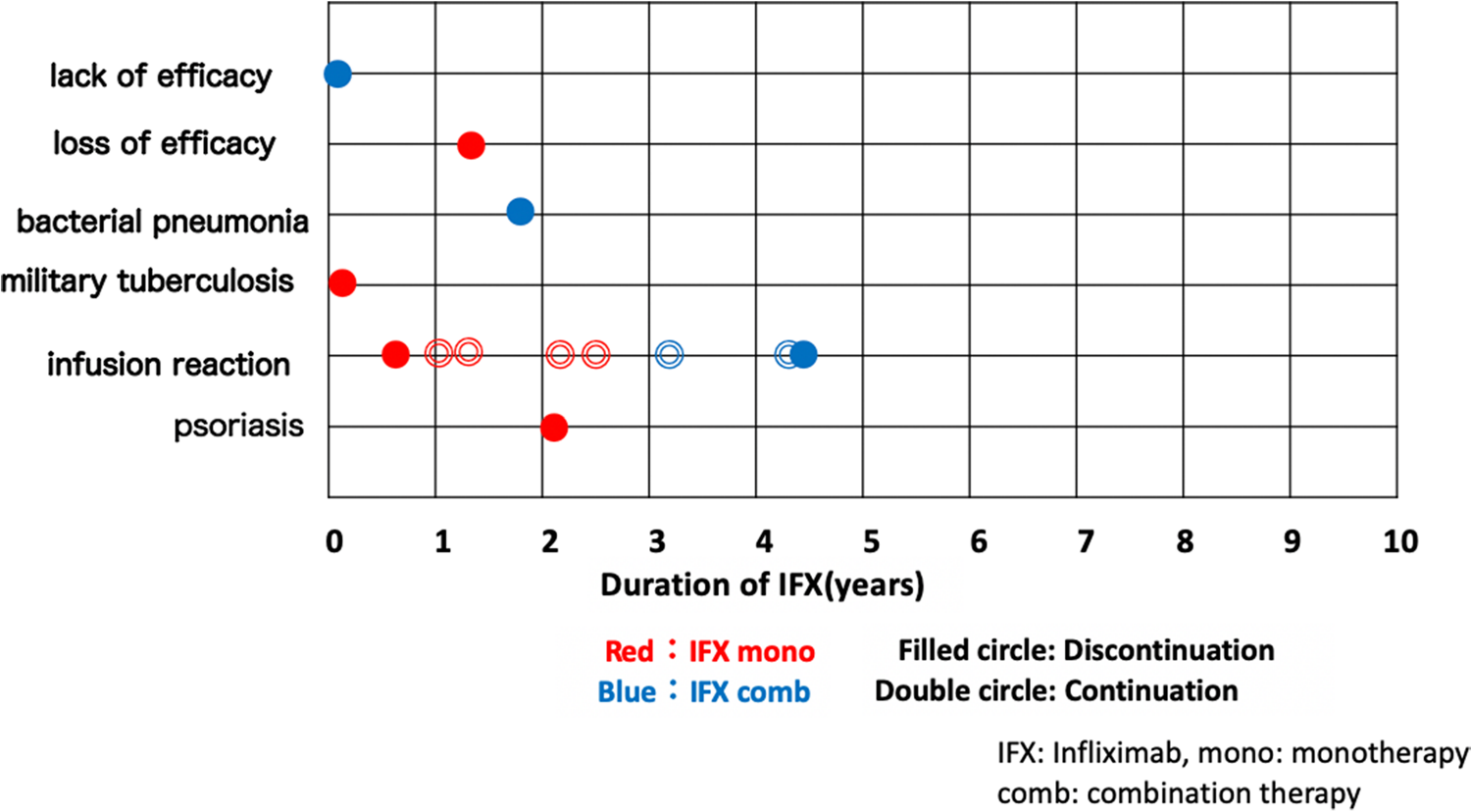
Occurrence and timing of adverse events. Filled circle shows the timing of the adverse events and the subsequent discontinuation of IFX. Double circle indicates the timing of AEs during the continuation of IFX. All of the AEs were seen during initial 5-year period, with no discontinuations or AEs noted after 6 years of continuous IFX.

Figure 5 presents information on the onset of AEs, IFX discontinuation, and the timing for the incidence of AEs. Although AEs were seen during the initial 5-year period, there were no discontinuations or AEs observed after 6 continuous years of IFX.

## Discussion

Anti-TNF-α antibody treatment has been reported to be effective in decreasing uveitis frequency and the burden of intraocular inflammation ^20–22^, including retinal vasculitis ^23^. Many studies have observed improvement in the visual prognosis after the application of IFX treatment in BS patients starting in 2007 ^6^. Nevertheless, evidence regarding its long-term effectiveness in the medical literature is scarce. In addition, clarification of the responses to IFX monotherapy in BS patients as compared to that for combination therapy can potentially provide useful information regarding the necessity of concomitant drugs. Thus, the present study might be of great benefit, as it highlights the characteristic of IFX.

As for the analyzed population of BS in the current study, the characteristics of BS in the enrolled subjects were similar to those of previous reports in terms of the more prevalent male population^24^, ocular characteristics, and systemic characteristics (Table 1) ^13,20,21,23^.

Overall, the IFX retention rate for BS patients in this study was 70.3% at the 10-year follow-up, which was higher than the 47.11% reported in a previous study ^13^. In addition, our detailed analysis also showed that the retention rate between 1 to 5 years was 76.0% (Fig. 1a), which was almost equal to that reported in the previous study (75.7%) ^13^ after 5 years of treatment. In contrast, the retention rate between 6 to 10 years in the present study was 97.4%, which was much higher than that previously reported (62.3%) ^13^. The major reason for the discontinuation in the previous study between 6 to 10 years was secondary inefficacy ^13^.

Due to drug restrictions in Japan, we have not previously co-administered immunosuppressant agents, such as azathioprine and methotrexate, as part of a combination therapy. However, we did not observe any difference between the IFX monotherapy and IFX combination therapy during the 10 years of treatment in this study (Fig. 1b). Furthermore, the 10-year retention rate for the present study was superior to that reported for a previous study in which immunosuppressant agents were co-administered in 60% of the enrolled patients ^13^. It is thought that anti-IFX-antibody production might be related to the secondary inefficacy that occurs during long-term IFX use. Furthermore, the concomitant use of immunosuppressant agents has the potential to reduce the development of anti-IFX antibodies. In our current study, however, this theory of the development of an anti-IFX antibody does not completely explain our current findings. Potential reasons why we did observe a high persistency rate of IFX during the 6–10 year period in the present study might be that this phenomenon is related to 1) differences associated with the racial susceptibility to IFX, 2) differences in the disease severity of enrolled patients between these studies, and 3) a difference in the sample size assessed, which was lower in our present study.

Results for the frequency of ocular inflammatory attacks leading to severe visual loss showed that there was a significant improvement of the frequency of ocular inflammatory attacks after a 1-year IFX treatment. Although there was a slight increase of the attacks observed at 4 years, these ocular inflammatory attacks tended to be suppressed thereafter, with no recurrence detected after 7 years of treatment in all of our enrolled cases (Fig. 2a). Furthermore, our comparison analysis between the monotherapy and combination therapy groups showed that these inflammatory attacks decreased in a time-dependent manner in the combination therapy group. However, the increase in these attacks observed for the IFX monotherapy for the overall analysis after 4 years (Fig. 2b) could have been due to the slight increase that occurred during year 4. One possible reason for the increased inflammatory attacks at year 4 might be due to a loss of efficacy and a secondary failure caused by the induction of an anti-IFX antibody. A previous study reported finding that an anti-IFX antibody was produced at 2 years after the administration of IFX in European BS patients ^25^. Therefore, these differences between our study and the previous study could potentially be explained by a delay in the production of anti-IFX antibody in the Japanese BS patients as compared to that found in the European population. This is supported by our finding that there was a slight increase in inflammatory attacks at the 6-year point as compared to that observed for the previous 5-year combination therapy (Fig. 2b). Our results indicated that the combination therapy contributed to the suppression of production of anti-IFX antibodies, thereby potentially delaying the secondary failure and maintaining the tolerability much longer than that observed for the monotherapy (Fig. 2b). However, this speculation might still need to be further evaluated, as the sample size in this study was still small, while the CS dosages were additionally not high enough to induce relevant immunomodulation. Another potential reason could perhaps be determined by clarifying whether patients who encountered relapses at year 4 might have also decided that they were no longer satisfied with their progress and thus, changed their treatment**.** One other possibility is that the less responsive patients were not removed until a later time point, thereby facilitating at least a partial reduction of the frequency.

In conjunction with the reduction of the ocular inflammatory attacks, we observed a significant improvement of BCVA during the 10-year follow-up period for both the monotherapy and combination therapy (Fig. 3a, 3b), which is consistent with that reported in a previous study ^13^. These results were thought to be related to the suppression of ocular inflammatory attacks, which was discussed above.

The CS-sparing effect of the IFX treatment was 77.5% during the 10-year observation period of this study (Fig. 4). This is similar to the rate that was reported in a previous study (79.1%) ^13^. The significant CS-sparing effects found in both our study and this previous study confirmed that IFX contributes to the cessation of CS use in BS-related uveitis, thereby potentially helping to reduce the side effects that are associated with CS administration, such as secondary cataract, secondary glaucoma, and diabetes.

In the present study, we found 11 cases of AEs in 10 patients out of all of the enrolled patients. The most frequent AE was the infusion reaction (8 cases), which occurred at a much higher rate than that reported in a previous study ^13^. During our treatments, CS was not administered during the time of the IFX infusion. Thus, this could have increased the infusion reactions that occurred during the follow-up period, and therefore, might reflect the effect of IFX by itself. With regard to the type of therapy, AEs were seen in 7 out of 16 (43.8%) during monotherapy and in 4 out of 8 (50%) in combination therapy with CS/COL during the initial 5-year treatment (Tables 4, 5, Fig. 5). These results demonstrate that the AE appearance rate was not changed due to the CS/COL co-administration. Therefore, this shows that the IFX monotherapy was not inferior to IFX co-administered with CS/COL with regard to the risk of discontinuation of IFX.

As for indication of IFX for BS patients, IFX tends to be administered after encountering ineffectiveness of other conventional anti-inflammatory agents. Although many studies have shown that IFX was effective for BS uveitis, there are several obstacles with regard to the usage of IFX as a first line treatment. First, there are many guidelines and drug restrictions that are dependent on the medical circumstances in a particular country. For example, the administration of IFX in Japan can only be considered after demonstrating the insufficiency of anti-inflammatory agents (COL, CS, or CsA). Another reason is the heavy financial burden of IFX for patients with BS with regard to the expensive payment cost as compared to conventional anti-inflammation agents. This is a similar situation in Europe where accessing expensive drugs is not easy due to the economic welfare of a country, even though the drugs have been shown to have much more efficacy with regard to suppressing inflammatory diseases ^26^ . However, there has been a considerable price reduction recently observed for anti-TNF agents over the past few years ^17^. Furthermore, it is expected that there will be easier economic access to IFX for the treatment of BS in the future.

In a previous study that compared IFX with another anti-TNF-α biologic, ADA, results demonstrated that there were similarities in the drug retention rate between ADA and IFX, thereby suggesting IFX non-inferiority against the already approved ADA for the treatment of non-infectious uveitis ^22^. Although we focused on IFX in the present study and did not analyze ADA for BS patients, our results did confirm that IFX proved to be effective for BS-related uveitis. However, a comparison between these two biologics in BS patients might be essential for determining additional information for this field.

In addition to the retrospective design, there are several other potential limitations that need to be considered for the present study. First, although the current data for this 10-year follow-up study contain useful information for ophthalmologists who are currently treating or thinking of treating BS patients with IFX, an increased number of enrolled patients will be needed in order to obtain much more precise information. Therefore, in the next step, further accumulation of 10-year follow-up data from multiple facilities is required. Second, constant examinations regarding the occurrence of anti-IFX antibody and the concentration of IFX in the blood will need to be evaluated, as this could potentially reveal specific reasons for discontinuations due to AEs. Third, owing to the circumstances regarding the use of biologics for BS in Japan ^14^, there are presently no patients who have previously used other biologics prior to being given IFX. In addition, a previous study has also shown that biologic-naïve patients are more likely to have a better response to IFX ^13^. Therefore, the present study was not able to evaluate the effect of previous biologic treatments in the current patients. Fourth, immunosuppressant agents were not given as part of the co-administered drug therapy or to any of our enrolled BS patients in this study. Thus, the effect of IFX when combined with immunosuppressant agents might provide additional useful information in the future.

In conclusion, IFX was effective for suppressing severe uveitis in BS patients when given after the occurrence of insufficient conventional anti-inflammatory therapies over a 10-year follow-up period. Although the use of IFX as a monotherapy was not inferior to the use of combination therapy, attention to AEs is required during the first 5 years of administration, with safe continuation expected for the later phase of these administrations. IFX should be considered as one of the best options for long-term treatment use in BS patients.

## Methods

The study protocol conformed to the tenets of the Declaration of Helsinki and was approved by the Ethics Committee of Tokyo Medical and Dental University. A waiver of informed consent was approved by the Ethics Committee of Tokyo Medical and Dental University due to the retrospective nature of the study. The diagnosis of BS was made in line with the established criteria by the Behçet’s Disease Research Committee of Japan ^10,27^. Systemic investigations and blood tests were used to rule out infectious uveitis associated with herpes, syphilis and toxoplasma prior to starting IFX. In addition, checkups for active or latent systemic infections, such as tuberculosis, hepatitis B, as well as heart failure, malignant tumor and other systemic diseases were conducted. Furthermore, work-ups with X-rays and urinalysis in conjunction with consulting physicians, who also evaluated all of the examined data, were done in order to confirm that initiation of IFX was possible.

After demonstrating conventional drug insufficiency when using COL, CS, or CsA, IFX treatment was initiated and utilized as second line therapy in all cases. The standard regimen used was as follows: 5 mg/kg body weight of IFX was administered at weeks 0, 2, and 6 and then every 8 weeks thereafter. At the time of the IFX infusion, simultaneous administration of CS during these infusions was never performed in any of the cases. After switching from conventional drugs to IFX, CsA was discontinued in all cases. The intervals for each infusion and the IFX dose were adjusted in accordance with the BS activity. Ophthalmic assessment was performed every 4–8 weeks. Serum biochemical and hematological profiles were monitored at each clinic visit.

This retrospective study reviewed the medical records of BS patients evaluated at the Tokyo Medical and Dental Hospital and who were started on IFX between September 2005 and December 2008. In order to be eligible for enrollment in the study, patients had to be diagnosed with BS and have recurrent active uveitis that was uncontrollable with conventional drugs (COL, CS, and CsA) and required IFX treatment. Patients were excluded if they had eyes with permanent blindness prior to the IFX treatment, as the effect of IFX would have been difficult to evaluate.

The primary aim of this study was to evaluate the retention rate of IFX in patients with BS-related uveitis during a 10-year follow-up period. The secondary aims were to identify (1) the potential impact of an adjunctive traditional immunosuppressant in the IFX retention rate, (2) any change in the visual acuity and frequency of inflammatory attacks between baseline and every annual visit point during a 10-year follow-up, (3) the CS-sparing effect of IFX, and (4) safety of IFX as well as the onset of AE during the treatment.

The primary endpoint was based on the evaluation of the Kaplan–Meier survival curve in all patients treated with IFX at every annual visit point during the 10-year follow-up period. The secondary endpoints were (1) log-rank test for differences in the survival curves between the monotherapy and combination therapy, (2) Wilcoxon signed-rank test for any statistically significant difference in the BCVA values (LogMAR was selected to calculate the average BCVA and standard deviation) and the frequency of inflammatory attacks between the baseline and every annual visit point during a 10-year follow-up, (3) Wilcoxon signed-rank test for the significant reduction in the mean prednisolone (or equivalent) dosage, and (4) the systemic adverse events recorded during the IFX treatment.

Statistical analysis for the IFX retention rate was performed using the Kaplan–Meier method and log-rank test. Wilcoxon signed-rank test was used to analyze the frequency of ocular inflammatory attack, BCVA value and prednisolone dosage. GraphPad prism (GraphPad Software, San Diego, CA, USA) was used for the statistical analysis.
